# Supplementary material for: MDM2-Mediated p21 Proteasomal Degradation Promotes Fluoride Toxicity in Ameloblasts
Source: Cells. 2019 May 10;8(5):436. doi: 10.3390/cells8050436 (PMC6562432; doi:10.3390/cells8050436)
Supplement: Supplementary file 1 [file cells-08-00436-s001.pdf]

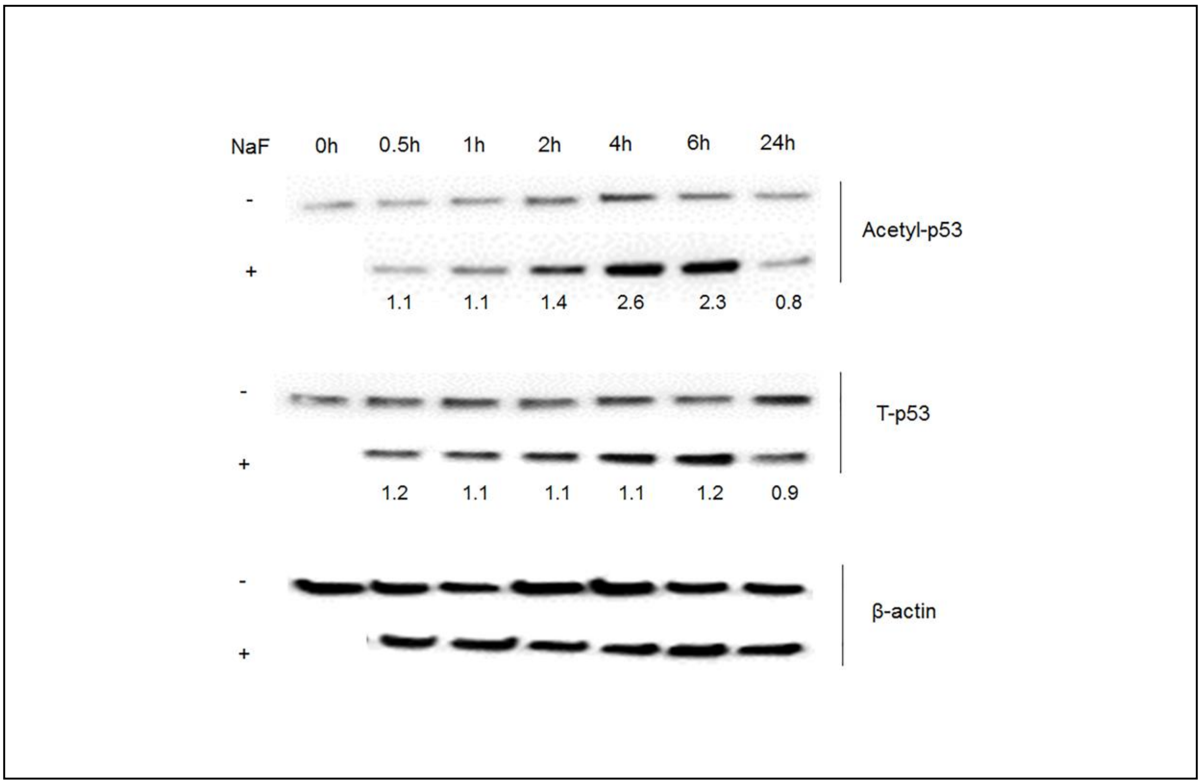

Supplementary Fig. S1. Fluoride induced formation of acetylated p53 (Ac-p53) in LS8 cells. LS8 cells were treated with NaF (5 mM) for 0-24 h. Ac-p53 (53 kDa) and total p53 (53 kDa) expression were detected by western blot. NaF treatment (+) increased Ac-p53 within 2-6 h. Fluoride treatment did not affect total p53 (T-p53) protein levels. The numbers show relative expression with NaF (+) versus without NaF (-) at each time point normalized by the loading control β-actin (44 kDa).

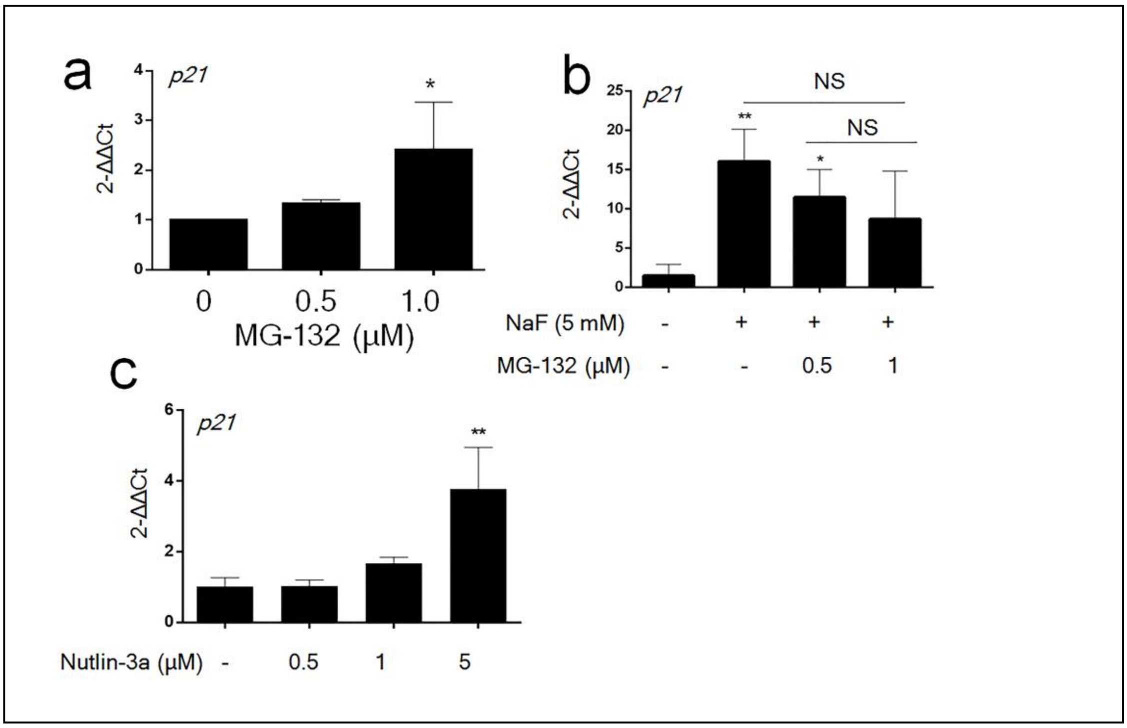

Supplementary Fig. S2. Effect of Nutlin-3a and MG-132 on *p21* mRNA expression in LS8 cells. LS8 cells were treated with (a) MG-132, (b) NaF and MG-132, (c) Nutlin-3a at the indicated concentrations for 24 h. *p21* mRNA was quantified by real-time qPCR. (a) MG-132 (1 μM) significantly increased *p21*

mRNA. (b) MG-132 did not alter fluoride-mediated *p21* mRNA expression. (c) Nutlin-3a (5 μM) significantly increased *p21* mRNA levels. Data are presented as means ± SD (**P* < 0.05 or ***P* < 0.01 vs Control).

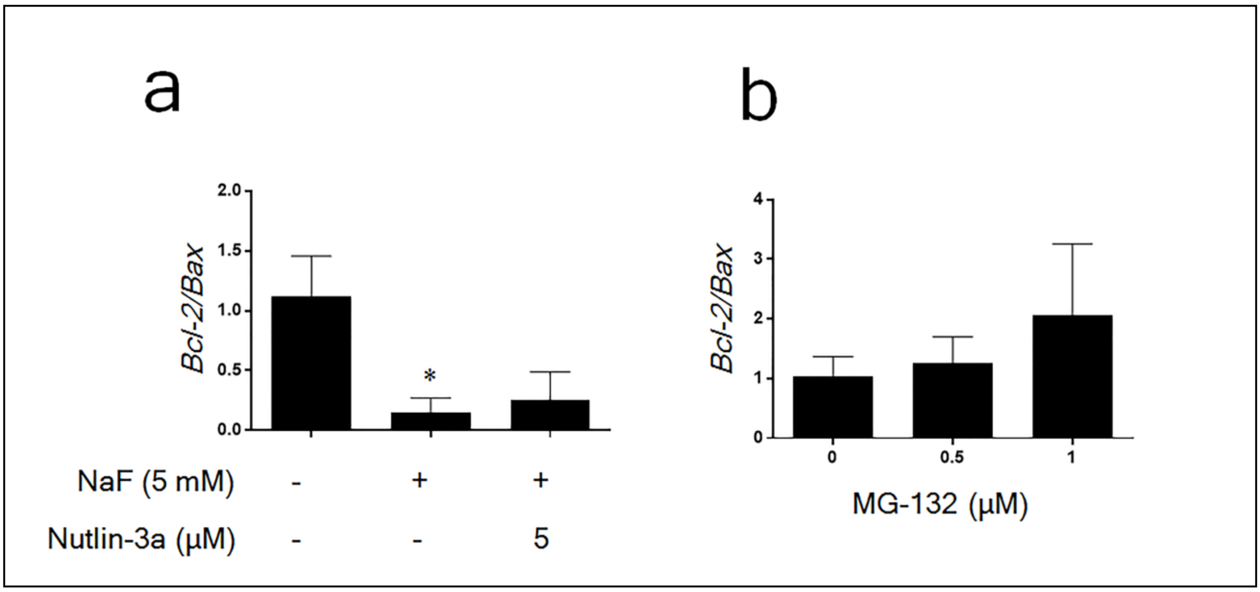

Supplementary Fig. S3. Effect of Nutlin-3a and MG-132 on *Bcl2/Bax* mRNA ratios in LS8 cells. LS8 cells were treated with (a) NaF (5 mM) with/without Nutilin-3a (5 μM) or (b) MG-132 (0.5-1.0 μM) for 24 h. The *Bcl-2/Bax* mRNA ratio was quantified by q-PCR. (a) NaF significantly decreased the *Bcl-2/Bax* mRNA ratio and this was not altered by Nutilin-3a treatment. (b) MG-132 treatment did not significantly increase the *Bcl-2/Bax* mRNA ratio. Data are presented as means ± SD. (**P* < 0.05 vs Control).

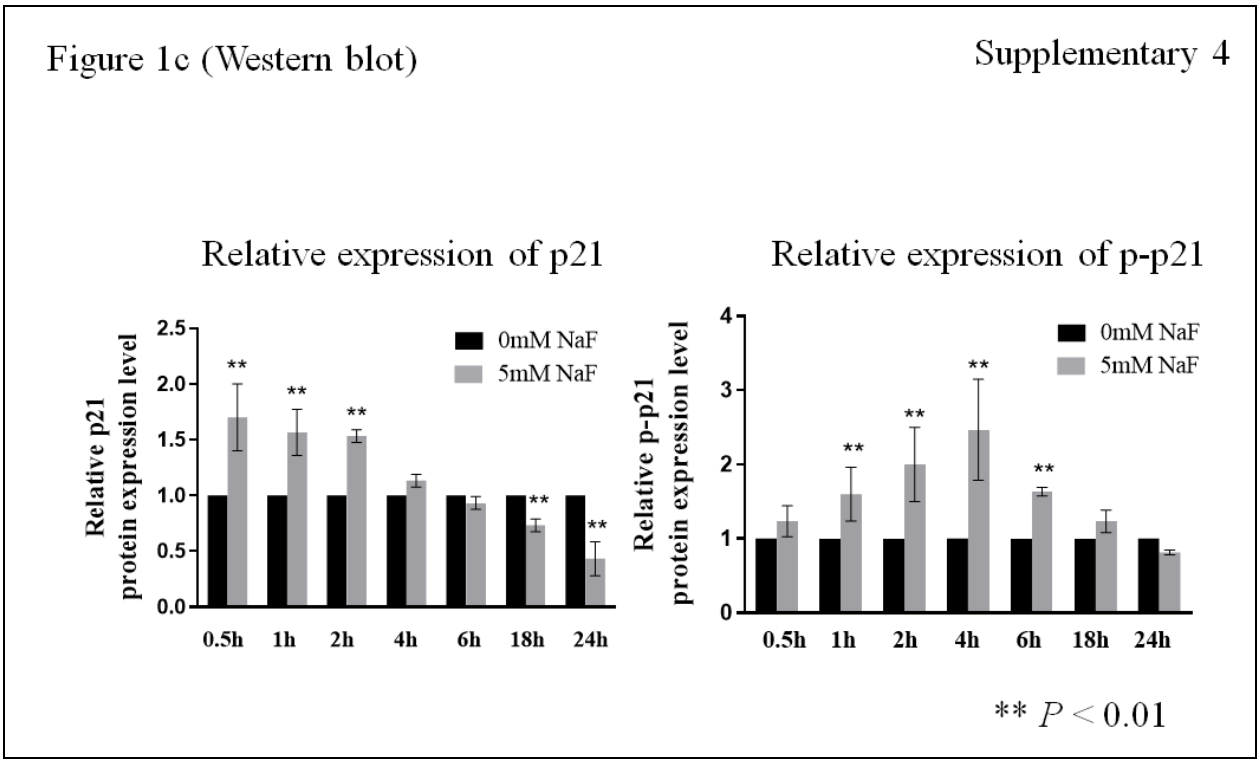

Supplementary Fig. S4. Statistical analysis of relative expression of p21 and p-p21 in LS8 cells. LS8 cells were treated with NaF (5 mM) for the indicated times and p21 (18 kDa) and p-p21 (21 kDa) were

detected by western blots. Protein expression was normalized by use of the loading control protein (β-actin). Relative protein expression and statistical significance were analyzed. Data are presented as means ± SD. (*$P < 0.05$, **$P < 0.01$ vs 0 mM NaF).

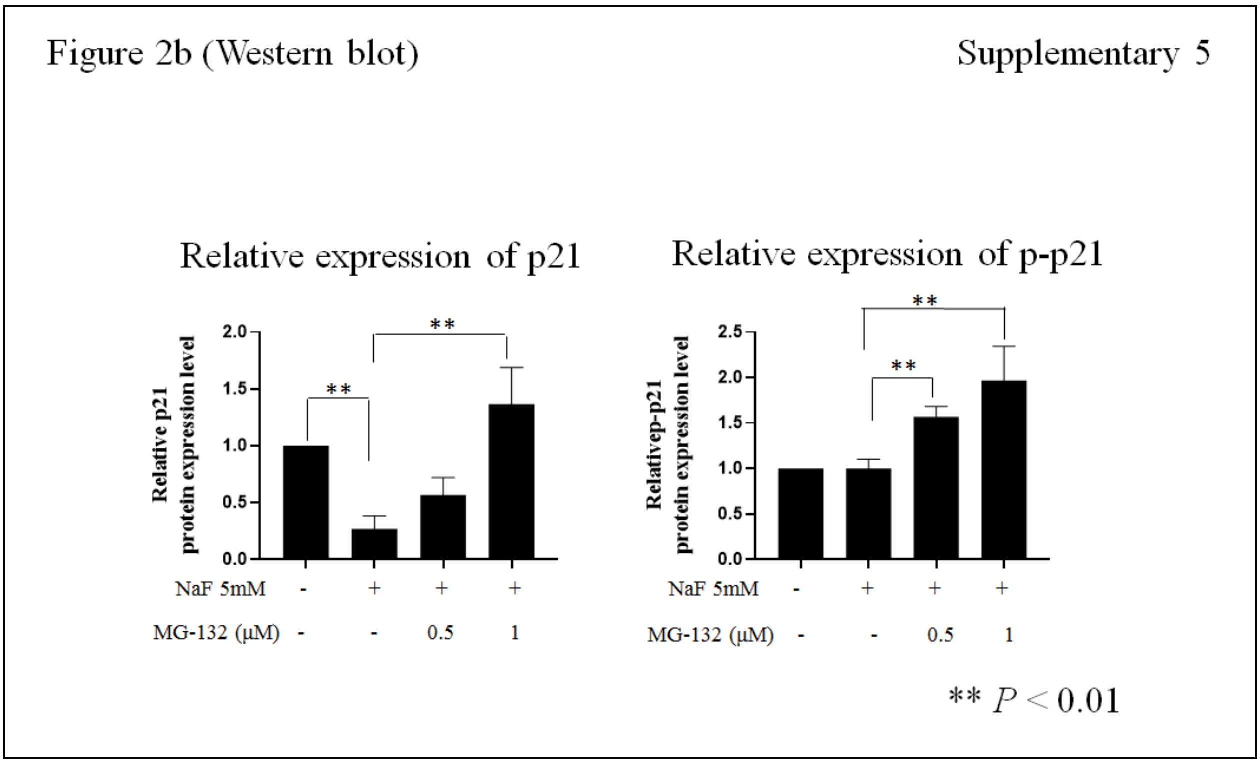

Supplementary Fig. S5. Statistical analysis of relative expression of p21 and p-p21 in LS8 cells. LS8 cells were treated with MG-132 (0.5-1.0 μM) for 2 h prior to NaF (5 mM) treatment for 24 h. p21 (18 kDa) and p-p21 (21 kDa) were detected by western blot. Protein expression was normalized by use of the loading control protein (β-actin). Relative protein expression and statistical significance were analyzed. Data are presented as means ± SD. (**$P < 0.01$).

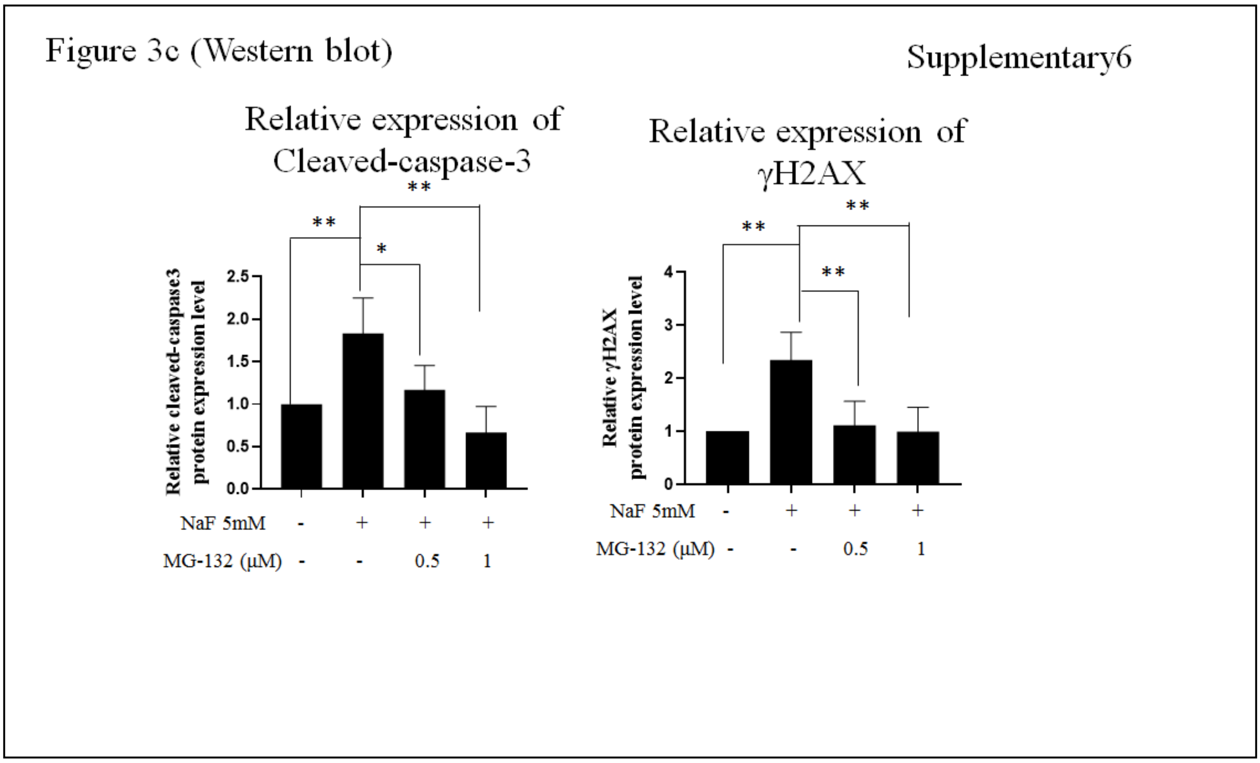

Supplementary Fig. S6. Statistical analysis of relative expression of Cleaved-caspase-3 and γH2AX in LS8 cells. LS8 cells were treated with MG-132 (0.5-1.0 μM) for 2 h prior to NaF (5 mM) treatment for 24 h. γH2AX (15 kDa) and cleaved-caspase-3 (17 kDa) were detected by western blots. Protein expression was normalized by use of the loading control protein (β-actin). Relative protein expression and statistical significance were analyzed. Data are presented as means ± SD. (**P* < 0.05, ***P* < 0.01).

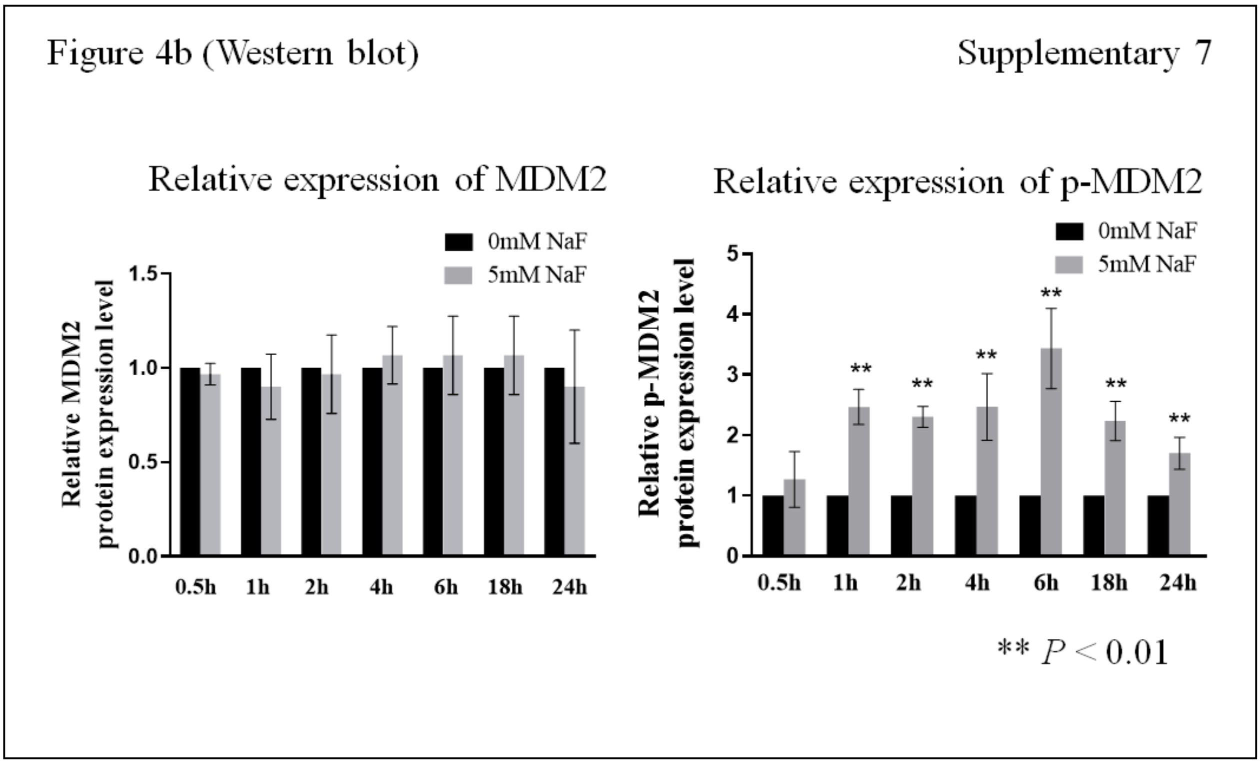

Supplementary Fig. S7. Statistical analysis of relative expression of MDM2 and p-MDM2 in LS8 cells. Cells were treated with NaF (5 mM) for the indicated times. Whole cell lysates were subjected to western blot analysis for phospho-MDM2 (p-MDM2 [Ser166]) (90 kDa) and total MDM2 (MDM2) (90 kDa) expression. Protein expression was normalized by use of the loading control protein (β-actin). Relative protein expression and statistical significance were analyzed. Data are presented as means ± SD. ***P* < 0.01 vs 0mM NaF).

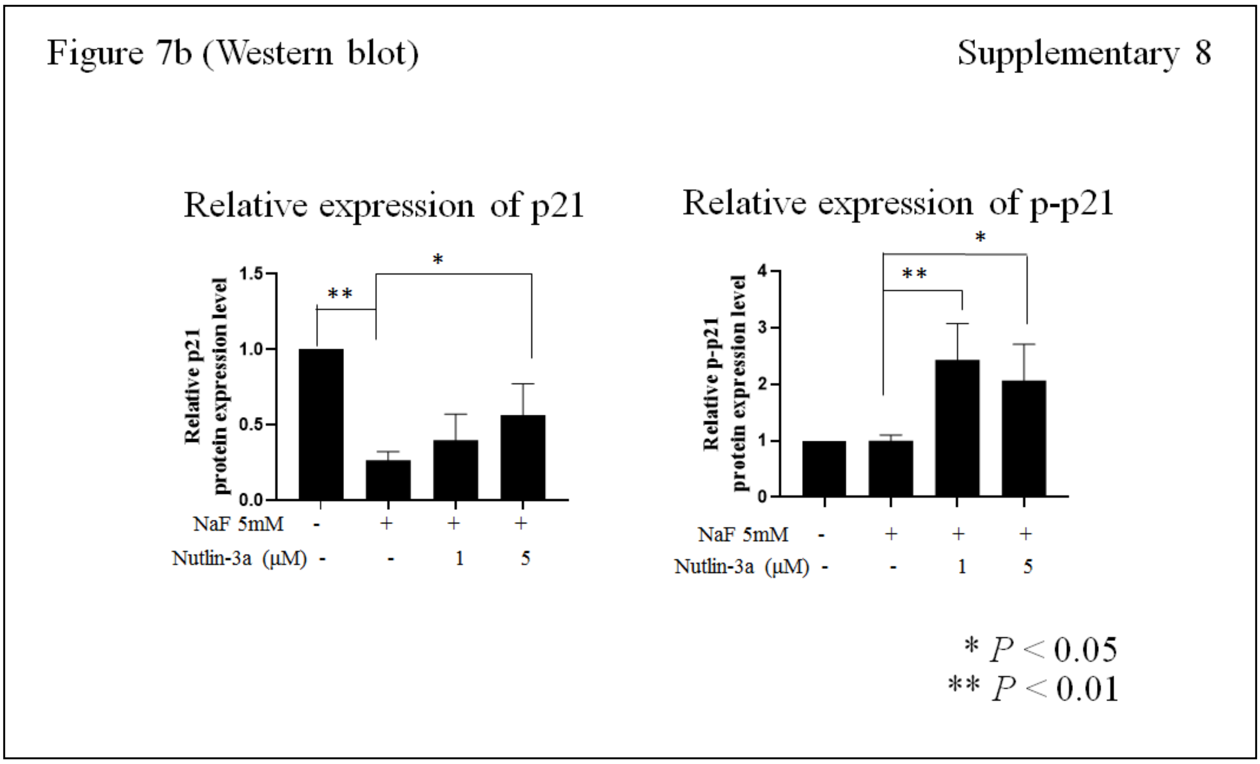

Supplementary Fig. S8. Statistical analysis of relative expression of p21 and p-p21 in LS8 cells. LS8 cells were treated with Nutlin-3a (1-5 μM) for 2 h followed by the additional NaF (5 mM) for 24 h. p21 (18 kDa) and p-p21 (21 kDa) were detected by western blots. Protein expression was normalized by use of the loading control protein (β-actin). Relative protein expression and statistical significance were analyzed. Data are presented as means ± SD. (*$P < 0.05$, **$P < 0.01$).

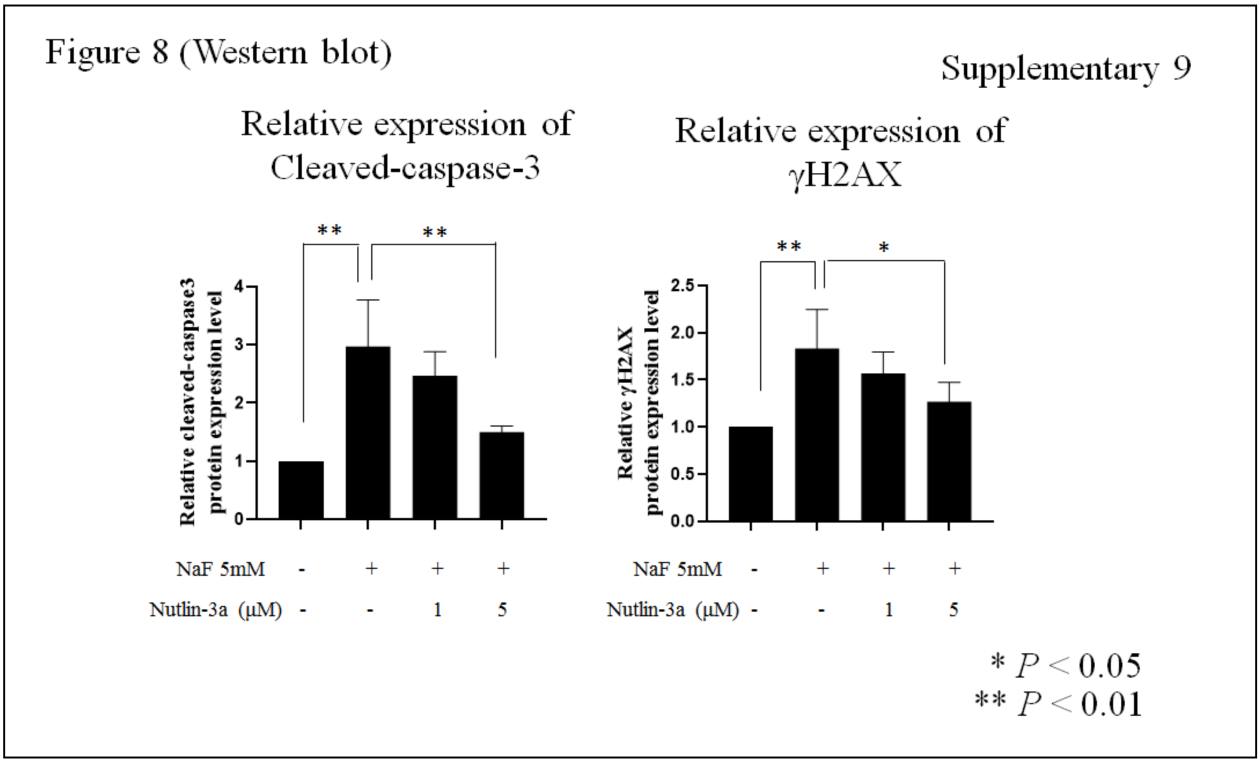

Supplementary Fig. S9. Statistical analysis of relative expression of cleaved-caspase-3 and γH2AX in LS8 cells. LS8 cells were treated with Nutlin-3a (1 μM or 5 μM) for 2 h followed by the addition of

NaF (5 mM) for 24 h. DNA damage marker γH2AX (15 kDa) expression and caspase-3 cleavage (17 kDa) were detected by western blot. Protein expression was normalized by use of the loading control protein (β-actin). Relative protein expression and statistical significance were analyzed. Data are presented as means ± SD. (*$P < 0.05$, **$P < 0.01$).

Table S1. Primers used for quantitative Real-time PCR.

**Gene**	**GenBank ID #**	**5′ Primer**	**3′ Primer**
p21	NM_007669.5	AATTGGAGTCAGGCGCAGAT	CGAAGAGACAACGGCACACT
Mdm2	NM_010786.4	GTCTGTGTCTACCGAGGGTG	TAAGTGTCGTTTTGCGCTCC
Bax	NM_007527.3	AGCTGCCACCCGGAAGAAGACCT	CCGGCGAATTGGAGATGAACTG
Bcl-2	NM_009741.5	TGGATGACTGAGTACCTGAACC	GCCAGGAGAAATCAAACAGAGG
Gapdh	NM_001289726	GCAAAGTGGAGATTGTTGCCAT	CCTTGACTGTGCCGTTGAATTT
